# Supplementary material for: Microbiome-based environmental monitoring of a dairy processing facility highlights the challenges associated with low microbial-load samples
Source: NPJ Sci Food. 2021 Feb 15;5:4. doi: 10.1038/s41538-021-00087-2 (PMC7884712; doi:10.1038/s41538-021-00087-2)
Supplement: Supplementary file 1 — SI [file 41538_2021_87_MOESM1_ESM.pdf]

Supplementary Figures

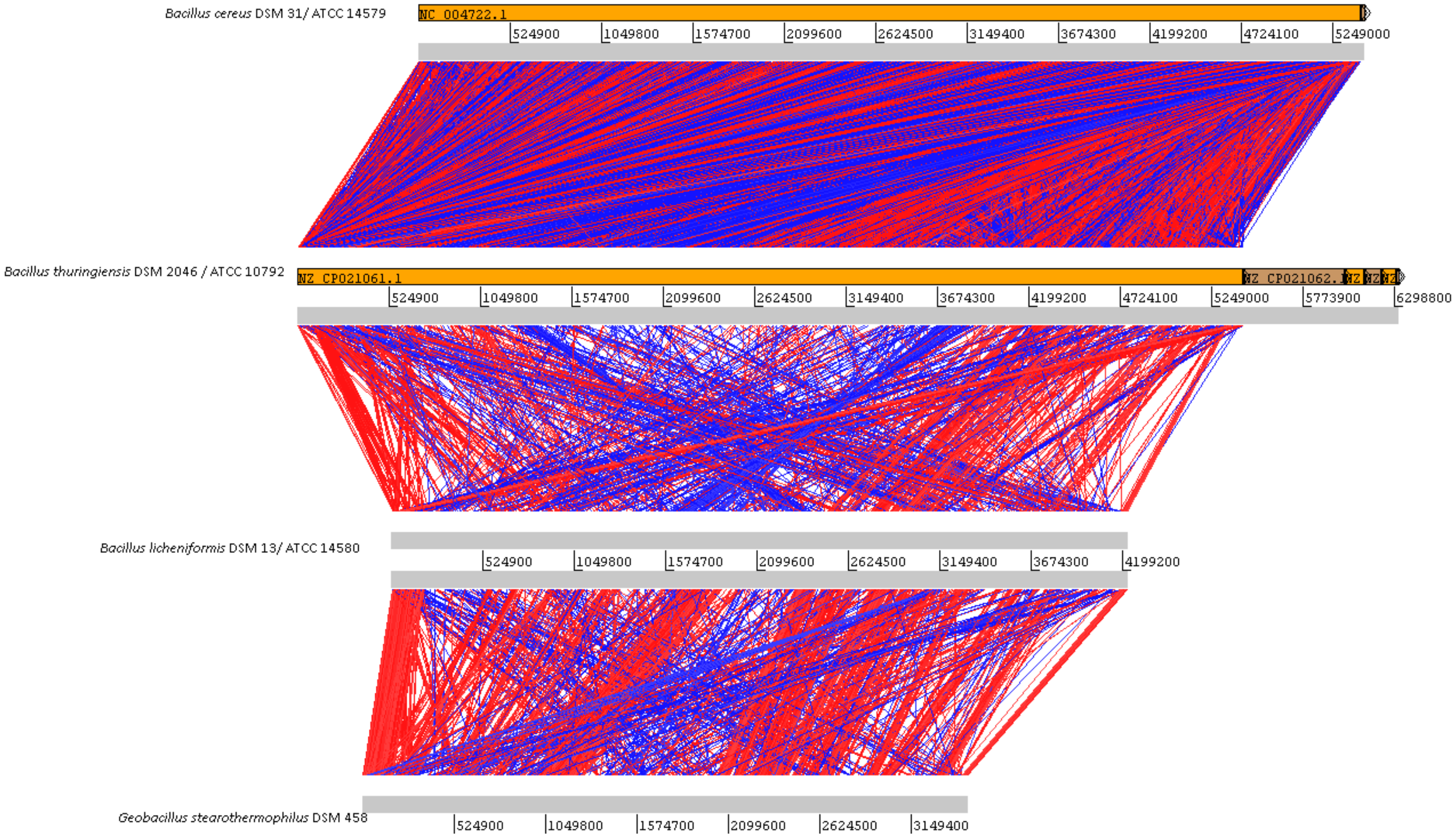

Supplementary Figure 1. Comparison of genomes of 4 strains used in mock community using ACT.

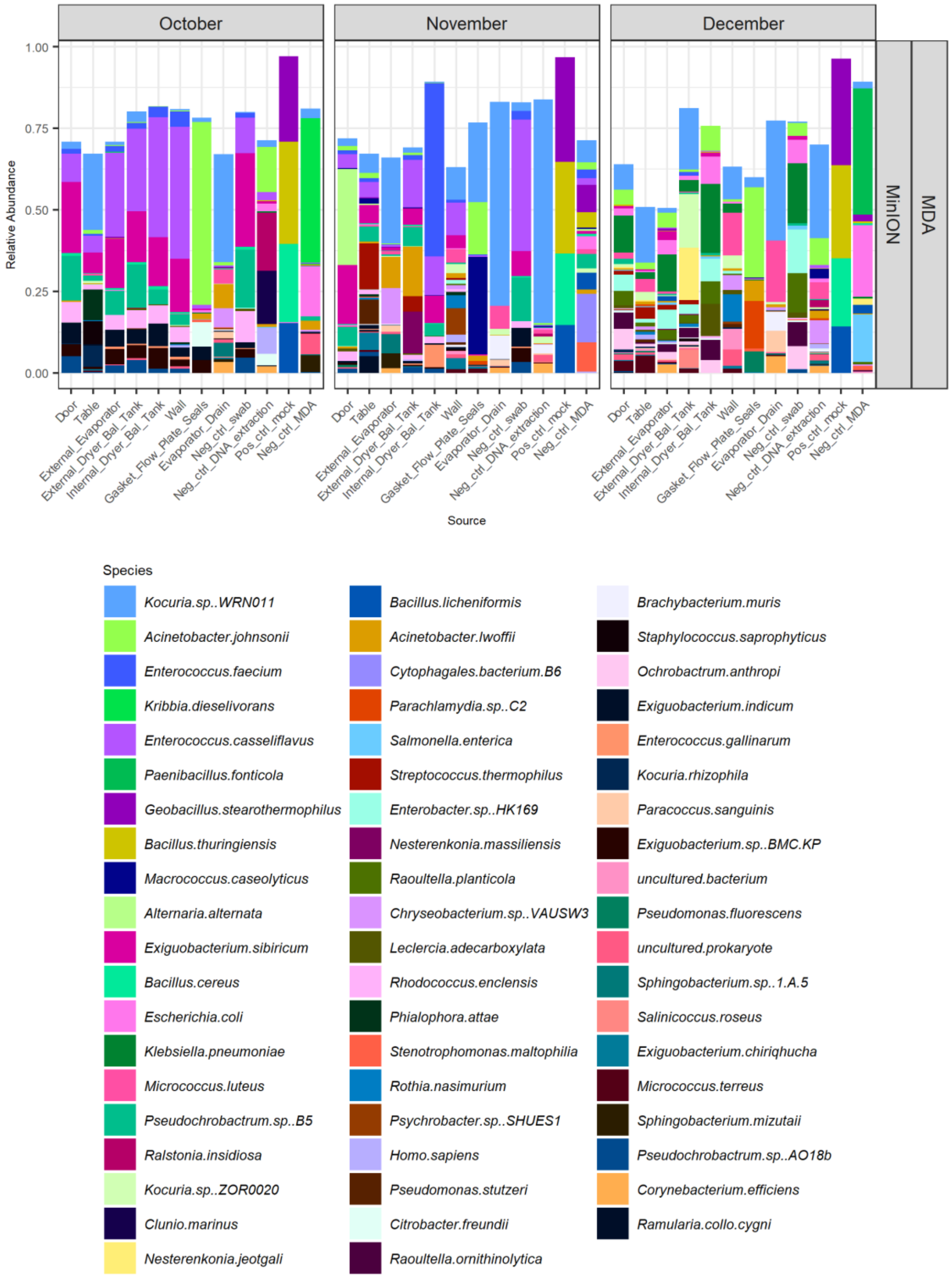

Supplementary Figure 2. Species level classification of MinION environmental samples.

Oxford Nanopore Technologies MinION sequencing of MDA DNA from environmental swab samples classified using LAST+MEGAN LR. Species present > 5% relative abundance in at least one sample are shown.

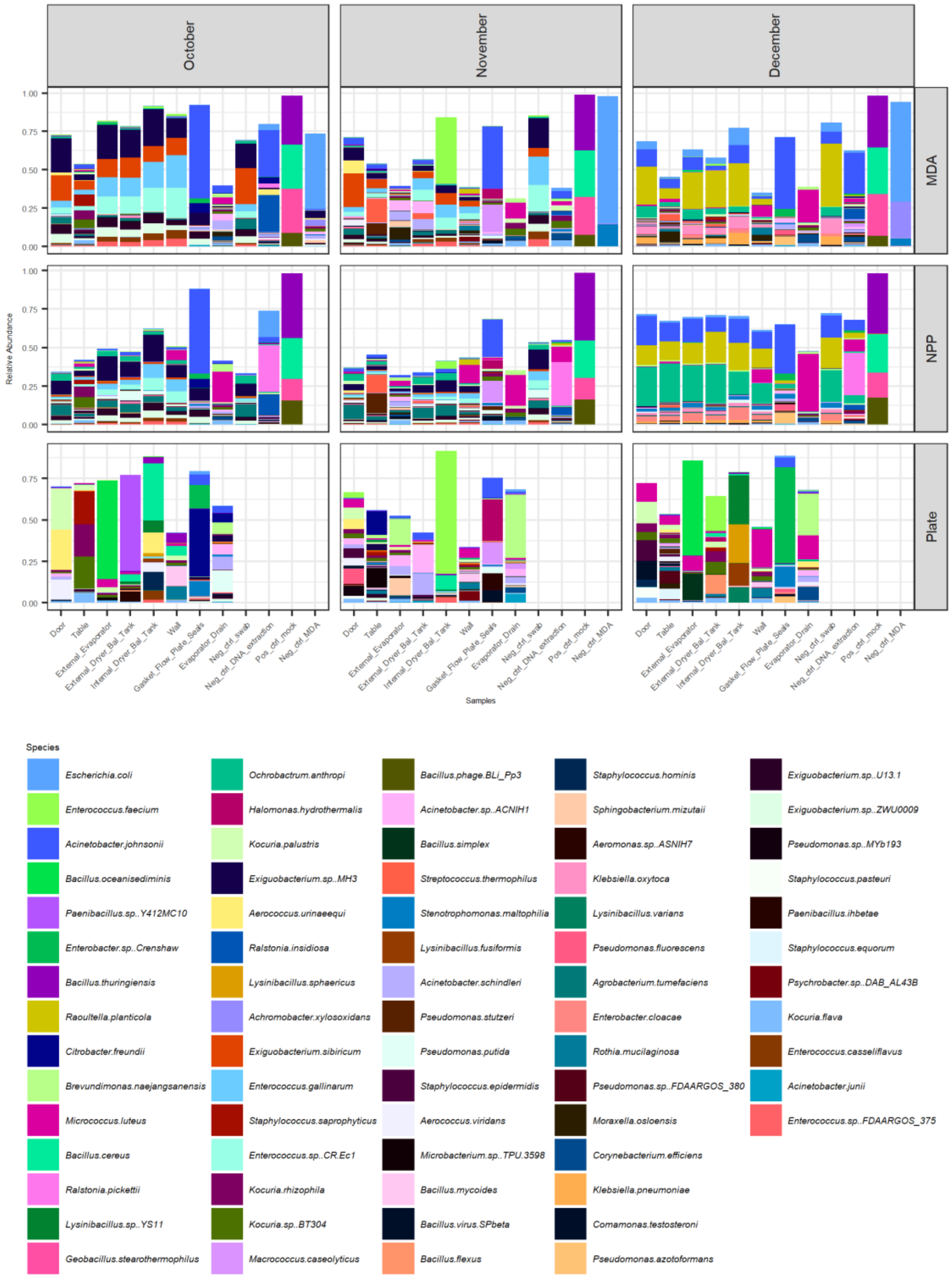

Supplementary Figure 3. Bracken classification of NextSeq sequences.

Kraken2 with Bracken species level classification of NextSeq samples. 100% mock community was classified, but yet could not accurately decipher species level classification.

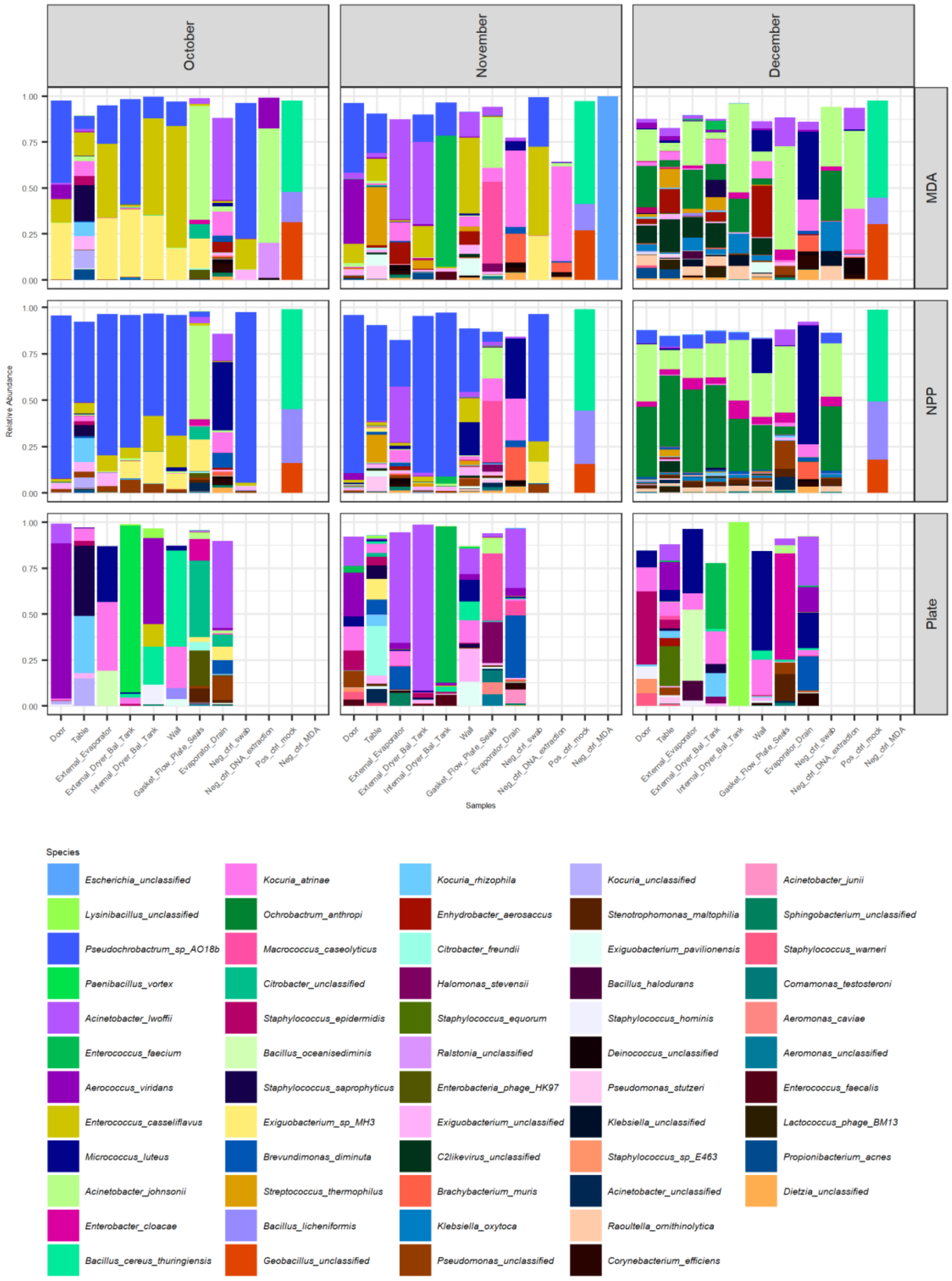

Supplementary Figure 4. MetaPhlAn2 species level classification of NextSeq data. MetaPhlAn2 species level classification of NextSeq data. Again highlights an incorrectly classified positive control/mock community.

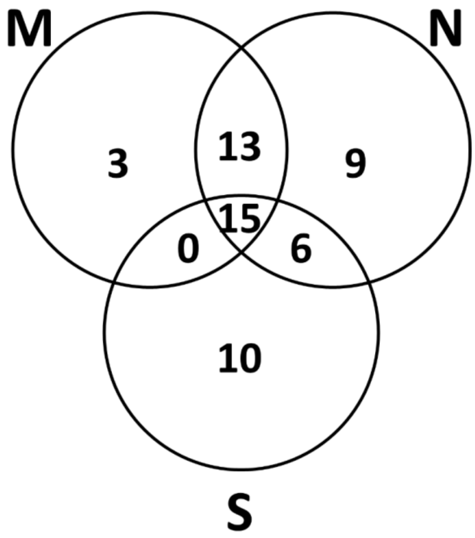

Supplementary Figure 5. Venn diagram of the number of genera assigned per sequence type.

Venn diagram shows number of genera assigned per sequencing type, MinION > 5% relative abundance (M), NextSeq > 5% relative abundance (N), Sanger (S).

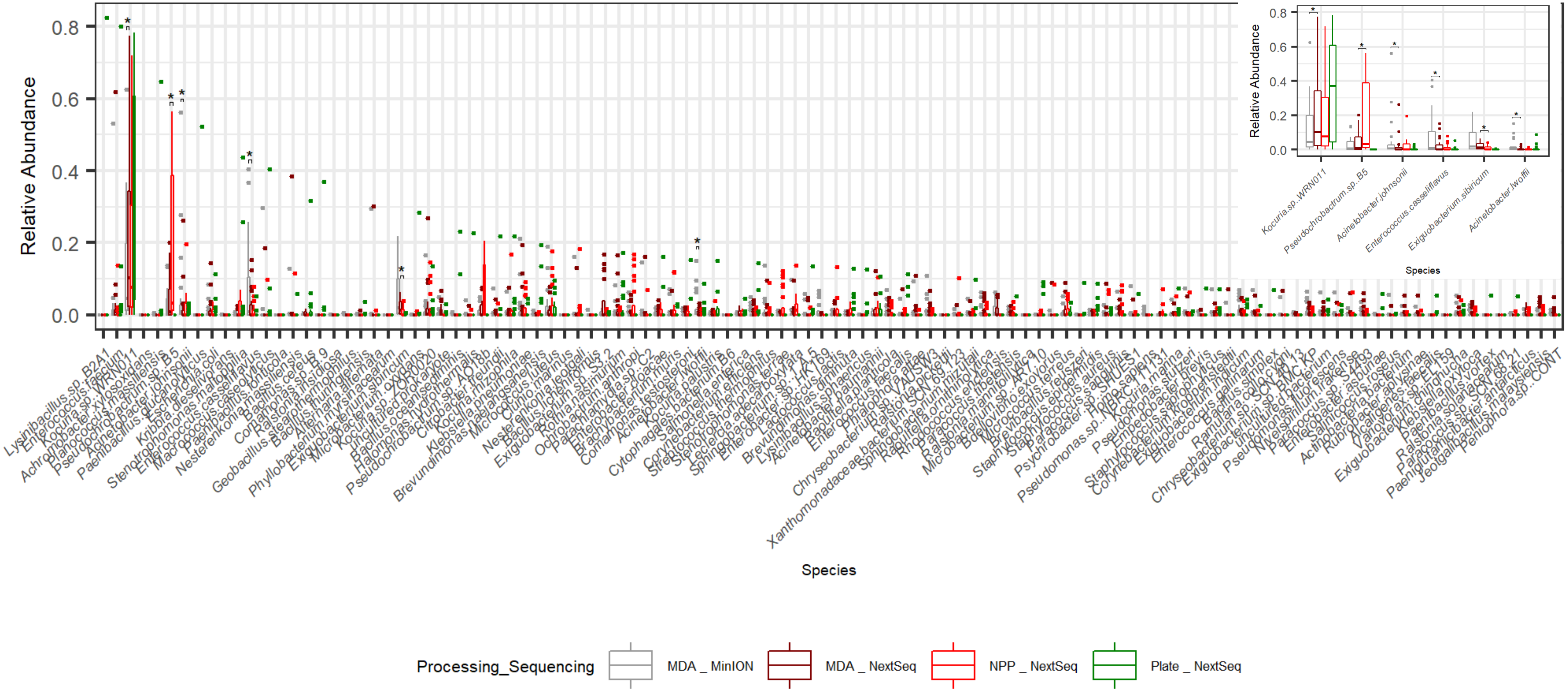

Supplementary Figure 6. Distribution of species level classification in environmental samples

Distribution of species classification by sequencer and pre-processing type. Significant differences highlighted. Controls not included in these, only environmental samples. Boxplot center line, median; box limits, upper and lower quartiles; whiskers, 1.5x interquartile range; points, outliers. (*** = $p < 0.001$, ** = $p < 0.01$, * = $p < 0.05$).

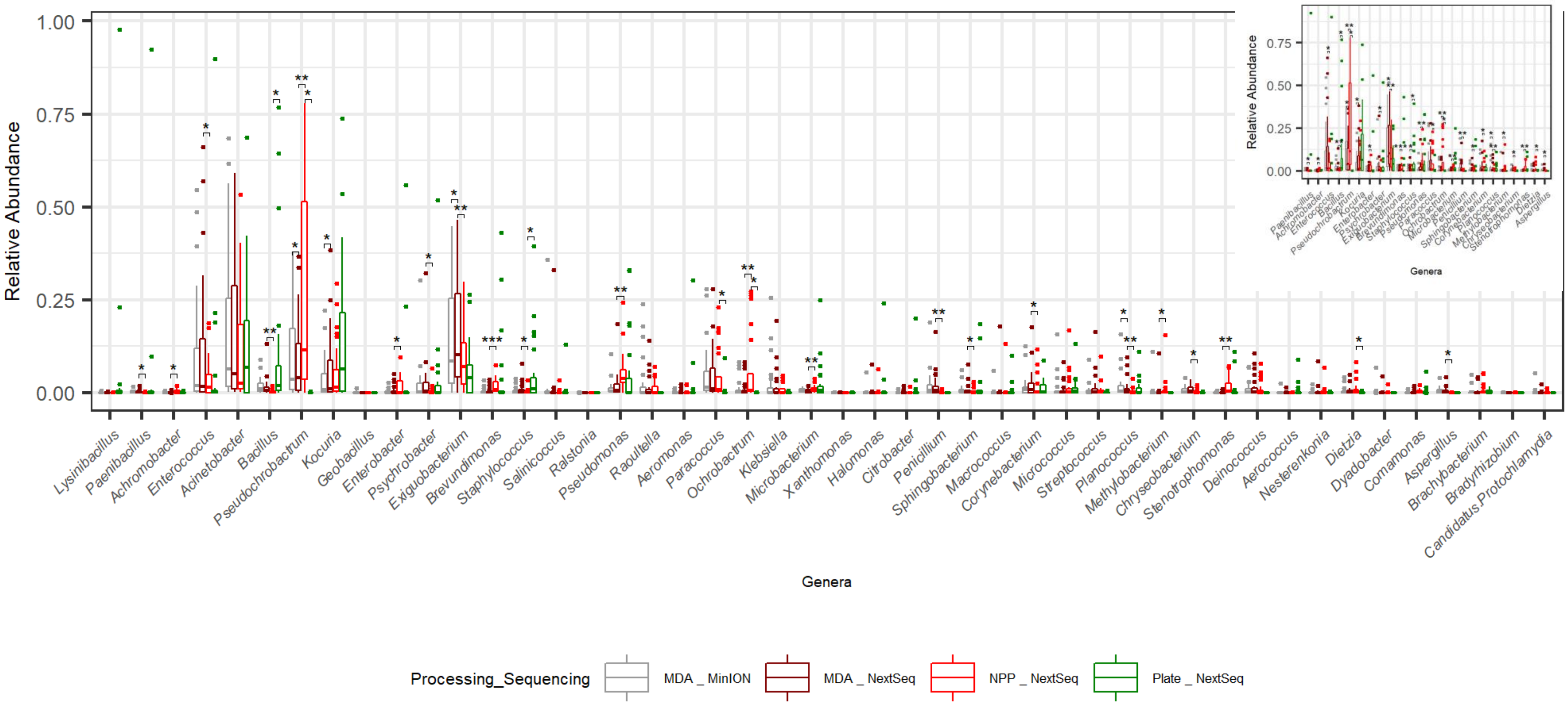

Supplementary Figure 7. Distribution of genera level classification in environmental samples.

Distribution of genera classification by sequencer and pre-processing type. Significant differences highlighted. Controls not included in these, only environmental samples. Boxplot center line, median; box limits, upper and lower quartiles; whiskers, 1.5x interquartile range; points, outliers. (*** = $p < 0.001$, ** = $p < 0.01$, * = $p < 0.05$).

Supplementary Tables

Supplementary Table 1. Alignment of sequenced mock metagenome assembled genomes (QRY) to reference genomes from NCBI RefSeq (REF), using MUMmer

	[REF]	[QRY]
[Sequences]		
TotalSeqs<br>AlignedSeqs<br>UnalignedSeqs	10<br>9(90.00%)<br>1(10.00%)	104<br>104(100.00%)<br>0(0.00%)
[Bases]		
TotalBases<br>AlignedBases<br>UnalignedBases	19432784<br>19095736(98.27%)<br>337048(1.73%)	17433642<br>17362744(99.59%)<br>70898(0.41%)
[Alignments]		
1-to-1<br>TotalLength<br>AvgLength<br>AvgIdentity	243<br>17330360<br>71318.35<br>97.94	243<br>17043215<br>70136.69<br>97.94

Supplementary Table 2. Counts of culturable bacteria from environmental samples and their classification

**Source**	**Month**	**Culture type**	**CFU / Sample**	**CFU / Swab**	**Genera**
Door	October	TBC	2.00E+04	4.00E+03	*Acinetobacter, Aerococcus, Aerococcus, Kocuria, Kocuria, Psychrobacter*
		ST	0.00E+00	0.00E+00	N/A
		SM	0.00E+00	0.00E+00	N/A
Table	October	TBC	3.34E+05	6.69E+04	*Aerococcus, Bacillus, Kocuria*
		ST	7.00E+01	1.40E+01	*Bacillus*
		SM	1.05E+04	2.10E+03	*Bacillus, Bacillus, Bacillus, Bacillus, Paenibacillus, Paenibacillus*
External Evaporator	October	TBC	5.88E+03	1.18E+03	*Bacillus, Lysinibacillus, Micrococcus*
		ST	3.50E+02	7.00E+01	*Bacillus*
		SM	2.94E+03	5.88E+02	*Bacillus, Bacillus, Bacillus*
External Dryer Bal Tank	October	TBC	9.38E+03	1.88E+03	*Bacillus, Bacillus, Bacillus, Chryseomicrobium, Exiguobacterium, Lysinibacillus*
		ST	7.00E+01	1.40E+01	N/A
		SM	7.00E+01	1.40E+01	N/A
Internal Dryer Bal Tank	October	TBC	3.22E+03	6.44E+02	*Bacillus, Lysinibacillus, Staphylococcus, Staphylococcus*
		ST	0.00E+00	0.00E+00	N/A
		SM	0.00E+00	0.00E+00	N/A
Wall	October	TBC	9.87E+03	1.97E+03	*Bacillus, Bacillus, Microbacterium, Rothia, Rothia*
		ST	7.70E+02	1.54E+02	*Bacillus*
		SM	1.82E+03	3.64E+02	*Oceanobacillus*
Gasket Flow Plate Seals	October	TBC	3.86E+08	7.71E+07	*Acinetobacter*
		ST	0.00E+00	0.00E+00	N/A
		SM	7.06E+04	1.41E+04	*Bacillus, Lysinibacillus*
Evaporator Drain	October	TBC	2.85E+08	5.70E+07	*Corynebacterium, Kocuria, Psychrobacter*
		ST	5.25E+02	1.05E+02	*Bacillus*
		SM	7.50E+04	1.50E+04	N/A
Neg ctrl Swab	October	TBC	7.00E+01	1.40E+01	*Staphylococcus*
		ST	0.00E+00	0.00E+00	N/A
		SM	0.00E+00	0.00E+00	N/A
Door	November	TBC	1.90E+04	3.81E+03	*Aerococcus, Bacillus, Kocuria, Staphylococcus, Staphylococcus, Staphylococcus, Staphylococcus, Staphylococcus*
		ST	3.15E+02	6.30E+01	*Bacillus*
		SM	3.15E+02	6.30E+01	*Bacillus*

Table	November	TBC	1.53E+05	3.06E+04	*Bacillus, Bacillus, Exiguobacterium, Exiguobacterium, Kocuria, Microbacterium*
		ST	2.80E+02	5.60E+01	N/A
		SM	1.05E+03	2.10E+02	*Bacillus, Lysinibacillus, Rothia*
External Evaporator	November	TBC	1.24E+07	2.48E+06	*Acinetobacter, Kocuria, Microbacterium, Microbacterium, Sphingobacterium*
		ST	7.00E+01	1.40E+01	*Bacillus*
		SM	3.57E+03	7.14E+02	*Bacillus, Bacillus, Bacillus, Kocuria*
External Dryer Bal Tank	November	TBC	9.28E+04	1.86E+04	*Acinetobacter, Bacillus, Bacillus, Chryseomicrobium, Paracoccus*
		ST	7.00E+01	1.40E+01	*Bacillus*
		SM	2.00E+03	3.99E+02	*Bacillus, Bacillus, Bacillus*
Internal Dryer Bal Tank	November	TBC	1.73E+04	3.46E+03	*Enterococcus, Enterococcus, Lysinibacillus*
		ST	0.00E+00	0.00E+00	N/A
		SM	1.26E+03	2.52E+02	*Bacillus*
Wall	November	TBC	2.18E+05	4.37E+04	*Kocuria, Kocuria, Lysinibacillus, Planococcus, Psychrobacter, Rothia, Rothia*
		ST	4.20E+02	8.40E+01	*Bacillus*
		SM	7.98E+03	1.60E+03	*Bacillus, Bacillus, Bacillus*
Gasket Flow Plate Seals	November	TBC	2.12E+07	4.24E+06	*Acinetobacter, Halomonas, Kocuria, Macrococcus*
		ST	7.00E+01	1.40E+01	*Bacillus*
		SM	5.85E+04	1.17E+04	*Bacillus, Lysinibacillus, Lysinibacillus*
Evaporator Drain	November	TBC	2.15E+09	4.30E+08	*Kocuria, Microbacterium, Planococcus*
		ST	1.47E+03	2.94E+02	*Bacillus, Bacillus*
		SM	3.53E+05	7.06E+04	*Bacillus, Bacillus, Corynebacterium*
Neg ctrl swab	November	TBC	2.10E+02	4.20E+01	*Bacillus, Kocuria, Rothia*
		ST	0.00E+00	0.00E+00	N/A
		SM	2.10E+02	4.20E+01	*Kocuria*
Door	December	TBC	1.61E+04	3.22E+03	*Arthrobacter, Micrococcus, Psychrobacillus, Psychrobacillus, Staphylococcus, Staphylococcus*
		ST	7.00E+01	1.40E+01	*Bacillus*
		SM	1.40E+02	2.80E+01	N/A
Table	December	TBC	2.10E+05	4.19E+04	*Bacillus, Kocuria, Rothia, Macrococcus, Rothia, Rothia, Staphylococcus*
		ST	6.30E+02	1.26E+02	*Bacillus*
		SM	8.40E+02	1.68E+02	N/A

External Evaporator	December	TBC	7.56E+03	1.51E+03	*Bacillus, Ornithinibacillus, Ornithinibacillus*
		ST	1.26E+03	2.52E+02	*Bacillus, Brevibacillus*
		SM	3.08E+03	6.16E+02	*Bacillus, Bacillus*
External Dryer Bal Tank	December	TBC	4.92E+04	9.84E+03	*Bacillus, Kocuria, Marinilactibacillus, Rothia, Salinicoccus*
		ST	3.15E+02	6.30E+01	*Bacillus*
		SM	3.50E+02	7.00E+01	N/A
Internal Dryer Bal Tank	December	TBC	7.00E+01	1.40E+01	*Lysinibacillus, Lysinibacillus*
		ST	0.00E+00	0.00E+00	N/A
		SM	7.00E+01	1.40E+01	*Bacillus*
Wall	December	TBC	2.17E+05	4.33E+04	*Aerococcus, Bacillus, Kocuria, Planococcus, Rothia*
		ST	1.05E+02	2.10E+01	*Thermoactinomyces*
		SM	1.47E+03	2.94E+02	*Oceanobacillus*
Gasket Flow Plate Seals	December	TBC	7.06E+07	1.41E+07	*Acinetobacter, Acinetobacter, Chryseobacterium*
		ST	8.40E+02	1.68E+02	*Bacillus*
		SM	4.57E+04	9.14E+03	*Bacillus, Bacillus, Exiguobacterium*
Evaporator Drain	December	TBC	2.94E+08	5.88E+07	*Aerococcus, Brevundimonas, Sphingobacterium*
		ST	8.72E+03	1.74E+03	*Brevibacillus*
		SM	1.17E+05	2.35E+04	*Bacillus, Bacillus*
Neg ctrl swab	December	TBC	1.40E+02	2.80E+01	*Bacillus*
		ST	0.00E+00	0.00E+00	N/A
		SM	7.00E+01	1.40E+01	*Bacillus*

Counts and classification of BHI cultured samples (TBC) and mesophilic and thermophilic spore selected BHI cultured samples (SM and ST) following isolation of morphologically different isolates and identification by sanger sequencing of 16S region.
