# Supplementary material for: Microbiome-based environmental monitoring of a dairy processing facility highlights the challenges associated with low microbial-load samples
Source: NPJ Sci Food. 2021 Feb 15;5:4. doi: 10.1038/s41538-021-00087-2 (PMC7884712; doi:10.1038/s41538-021-00087-2)
Supplement: Supplementary file 2 — nr-reporting-summary [file 41538_2021_87_MOESM2_ESM.pdf]

Corresponding author(s): Paul Cotter

Last updated by author(s): Dec 8, 2020

Reporting Summary

Nature Research wishes to improve the reproducibility of the work that we publish. This form provides structure for consistency and transparency in reporting. For further information on Nature Research policies, see our Editorial Policies and the Editorial Policy Checklist.

Statistics

For all statistical analyses, confirm that the following items are present in the figure legend, table legend, main text, or Methods section.

n/a	Confirmed	
☐	☒	The exact sample size (n) for each experimental group/condition, given as a discrete number and unit of measurement
☒	☐	A statement on whether measurements were taken from distinct samples or whether the same sample was measured repeatedly
☐	☒	The statistical test(s) used AND whether they are one- or two-sided<br>*Only common tests should be described solely by name; describe more complex techniques in the Methods section.*
☐	☒	A description of all covariates tested
☐	☒	A description of any assumptions or corrections, such as tests of normality and adjustment for multiple comparisons
☐	☒	A full description of the statistical parameters including central tendency (e.g. means) or other basic estimates (e.g. regression coefficient) AND variation (e.g. standard deviation) or associated estimates of uncertainty (e.g. confidence intervals)
☒	☐	For null hypothesis testing, the test statistic (e.g. F, t, r) with confidence intervals, effect sizes, degrees of freedom and P value noted<br>*Give P values as exact values whenever suitable.*
☒	☐	For Bayesian analysis, information on the choice of priors and Markov chain Monte Carlo settings
☒	☐	For hierarchical and complex designs, identification of the appropriate level for tests and full reporting of outcomes
☒	☐	Estimates of effect sizes (e.g. Cohen's d, Pearson's r), indicating how they were calculated

Our web collection on statistics for biologists contains articles on many of the points above.

Software and code

Policy information about availability of computer code

Data collection	Genome sequences for the 4 strains represented in the mock metagenomic community were downloaded from NCBI RefSeq. Bacillus cereus DSM 31/ATCC 14579, Bacillus thuringiensis DSM 2046/ATCC 10792, Bacillus licheniformis DSM 13/ATCC 14580 and Geobacillus stearothermophilus DSM 458 (Accession numbers GCF_000007825.1, GCF_002119445.1, GCF_000011645.1 and GCF_002300135.1, respectively)
Data analysis	Mock Community analysis:16S DNA sequences were rebasecalled using Albacore (version 2.2.6). FastQC was used to check sequence length and quality. IDBA fq2fa was used to convert fastq files to fasta format. BLASTn alignment of sequences against 16S Silva database (release 132) was performed with taxonomic classification by MEGAN (version 6.12.3). Genus and species levels of classification were determined, and relative abundances calculated and plotted using R ggplot2. Following basecalling with Albacore, Porechop (version 0.2.4) was used to remove adaptors from rapid WMGS reads before FastQC was used to check sequence length and quality and IDBA fq2fa was used to convert fastq format to fasta format. LAST alignment of reads was performed against the NR database (March 2018) with MEGAN long read (LR) (MEGAN version 6.12.3) taxonomic classification. Ranks were split, relative abundances calculated and plotted using R ggplot2.<br>The assembly of contigs from metagenomic reads was performed using Canu version 1.7 with -nanopore-raw flag. MUMmer alignment was performed on the assembled contigs against the 4 known species genomes from RefSeq, with dnadiff used to highlight differences between assemblies and reference genomes. The resulting comparisons were visualised using R ggbio and GenomicRanges.<br><br>Oxford nanopore sequenced environmental samples analysis:<br>Guppy basecalled reads obtained from MinKnow (version 18.12.4) were demultiplexed using Guppy barcoder version (2.1.3) to produce a barcoding summary text file. This contained the percentage match of each read to their barcodes with a minimum score of 60, the default). All fastq files produced by MinKnow were concatenated and guppy_bcsplit.py (https://github.com/ms-gx/guppy_bcsplit) allowed demultiplexing of reads based on their barcode assigned in the barcoding summary text file. Porechop (version 0.2.4) was used to remove adaptors from rapid kit sequence reads before Fastqc was used to check sequence length and quality. IDBA fq2fa was used to convert fastq to fasta. LAST alignment of fasta files against the NR database (March 2018) was performed with the MEGAN LR classification (MEGAN version 6.12.3). Files

were merged, ranks were split, total number of bases sequenced, and classified were calculated. Relative abundances calculated and plotted using R ggplot2.

Illumina sequenced environmental samples analysis:

BCl2fastq was used to convert raw sequence reads from Illumina NextSeq to fastq format. Kneaddata from bioBakery used trimmomatic for quality filtering and trimming paired end files with BMTagger to remove human and bovine reads. FastQC was used to visualise sequence length and quality. IDBA converted fastq to fasta. Diamond alignment of fasta files was performed against the NR database (march 2018) with MEGAN classification (MEGAN version 6.12.3). Files were merged, ranks were split, total number of bases sequenced and classified calculated and relative abundances calculated and plotted using R ggplot2.Illumina data was also analysed using Kraken2 and Bracken for taxonomy classification as well as using MetaPhlan2 for taxonomy classification for the purpose of comparison.

MDA amplified sequences from both Illumina and Oxford Nanopore sequencing were assembled using OPERA-MS. Illumina reads were then mapped against assemblies using bowtie2 and bam files sorted using samtools. Depth was calculated and Metabat2 ran on assembled contigs to produce bins. Checkm was used to determine the quality of the metagenome assembled genomes (MAGs). Prokka was used to generate .ffn files from bins, Kaiju-based taxonomic classification was performed on the open reading frames from prokka. Megan LR was also used on the whole bins for taxonomic classification of high quality MAGs.

For manuscripts utilizing custom algorithms or software that are central to the research but not yet described in published literature, software must be made available to editors and reviewers. We strongly encourage code deposition in a community repository (e.g. GitHub). See the Nature Research guidelines for submitting code & software for further information.

Data

Policy information about availability of data

All manuscripts must include a data availability statement. This statement should provide the following information, where applicable:

- Accession codes, unique identifiers, or web links for publicly available datasets
- A list of figures that have associated raw data
- A description of any restrictions on data availability

Sequence data have been deposited in the European Nucleotide Archive (ENA) under the study accession number PRJEB39267.

Field-specific reporting

Please select the one below that is the best fit for your research. If you are not sure, read the appropriate sections before making your selection.

☐ Life sciences ☐ Behavioural & social sciences ☒ Ecological, evolutionary & environmental sciences

For a reference copy of the document with all sections, see nature.com/documents/nr-reporting-summary-flat.pdf

Ecological, evolutionary & environmental sciences study design

All studies must disclose on these points even when the disclosure is negative.

Study description	Swabs were taken to study the microbiota of an active food processing facility. These were subject to culture based analysis as well as Microbiome analysis with Oxford Nanopore and Illumina-based sequencing. All results were compared. Eight locations in a single processing facility were swabbed on three different days across October, November, and December 2018, each after cleaning in place (CIP) but before the next round of dairy processing. These eight locations comprised a table, door, wall, gaskets/flow plate seals, external surface of dryer balance tank, internal surface of dryer balance tank, external surface of evaporator, and drain beside evaporator. These swabs were prepared for sequencing, along with a series of negative controls and a positive control, consisting of the simple mock metagenomic community.
Research sample	environmental swabs of a dairy processing facility
Sampling strategy	Environmental swabbing was performed in a commercial dairy processing pilot plant. Eight locations were swabbed during the course of a single day, after cleaning in place (CIP) had been completed and before the next round of dairy processing. These eight locations included a table, door, wall, gaskets/flow plate seals, external surface of dryer balance tank, internal surface of dryer balance tank, external surface of evaporator, and drain beside evaporator. Overall, these eight locations were swabbed over three different months (October, November, December), at a frequency of once per month.
Data collection	Swabs were taken by AM and MY and brought back to the lab for processing
Timing and spatial scale	Samples were taken on one day in each month of October, November and Decemeber 2018.
Data exclusions	N/A
Reproducibility	Swabs were taken from the same locations in 3 separate months, multiple controls were included in analysis including negative swab controls for each sampling day
Randomization	All samples were subject to multiple different sequencing methods and culture based analysis
Blinding	Blinding was not possible, as one person performed all swabbing, dna extraction and analysis

Did the study involve field work? ☐ Yes ☒ No

Reporting for specific materials, systems and methods

We require information from authors about some types of materials, experimental systems and methods used in many studies. Here, indicate whether each material, system or method listed is relevant to your study. If you are not sure if a list item applies to your research, read the appropriate section before selecting a response.

Materials & experimental systems

n/a	Involved in the study
☒	☐ Antibodies
☒	☐ Eukaryotic cell lines
☒	☐ Palaeontology and archaeology
☒	☐ Animals and other organisms
☒	☐ Human research participants
☒	☐ Clinical data
☒	☐ Dual use research of concern

Methods

n/a	Involved in the study
☒	☐ ChIP-seq
☒	☐ Flow cytometry
☒	☐ MRI-based neuroimaging
